# Myeloid Neoplasms in the Guise of Nutritional Deficiency

**DOI:** 10.1155/2012/826939

**Published:** 2012-11-25

**Authors:** Veda Parthasarathy

**Affiliations:** Department of Pathology, ESI-PGIMSR, 10 Sri Devi Krupa, 1st Main, 1st Block, RT Nagar, Bangalore 560032, India

## Abstract

The classic *BCR-ABL-*negative myeloproliferative neoplasms (MPNs) which include polycythemia vera (PV), essential thrombocythemia (ET), and primary myelofibrosis (PMF) are among the most frequent hematologic neoplasms. Because of their relatively smooth clinical course, it is likely that many of these MPNs actually go undetected. Considering the high prevalence of iron, folic-acid, and vitamin B_12_ deficiencies in developing countries, their coexistence with MPN can be expected frequently. In such situations where both disorders coexist, MPN is often overlooked. This causes considerable diagnostic delay. In this paper, two cases of PMF and one case of PV where the diagnosis of MPN was delayed for about 3 years are discussed. Presence of concomitant vitamin B_12_, folate, and iron deficiencies perhaps camouflaged the underlying MPN. Bearing in mind the possibility of MPN, even in the setting of apparent nutritional deficiency and performing a bone marrow evaluation, is the crucial step in unveiling the hidden MPN.

## 1. Introduction 

 Myeloproliferative neoplasms (MPNs) are among the most frequent hematologic neoplasms, usually affecting the middle aged and elderly [1]. “*Classic BCR-ABL-negative (Philadelphia negative) MPN*” is an operational subcategory [2, 3] which includes polycythemia vera (PV), essential thrombocythemia (ET), and primary myelofibrosis (PMF). Because of their relatively smooth clinical course, it is likely that many *classic BCR-ABL negative *MPN cases actually go undetected. Deficiencies of iron, folic acid, and Vitamin B_12_ are known to occur in association with *BCR-ABL-negative *MPNs [4–6]. These deficiencies may be coincident, secondary, or perhaps a manifestation of the underlying MPN.

 In developing countries where nutritional deficiencies are prevalent, their coexistence with MPN can be expected more often. In such situations where both disorders coexist, MPN often goes unnoticed. This paper illustrates three cases of BCR*-ABL-negative *MPN which went unnoticed for few years owing to concurrent nutritional deficiencies. 

## 2. Case 1

A 24-year-old female was referred with six months history of weakness and anorexia. Her records revealed that she had received hematinics and red cell concentrates several times in the past two years. She had undergone upper GI endoscopy and colonoscopy which were unremarkable. She was moderately built, had mild icterus, and spleen was palpable 4 cms below left costal margin. Her blood counts: Hb 9.3 g/dL, hematocrit 33.1%, WBC 8.8 × 10^9^/L, platelets 682 × 10^9^/L, MCV 103.8 fl, MCH 29.2 pg, mean platelet volume (MPV) 12.2 fl, RBC 3.2 × 10^12^/L, and reticulocytes 4%. Serum total bilirubin was 3.2 mg/dL, largely unconjugated and liver enzymes within normal range. Serum vitamin B_12_ was 107 pg/mL and LDH 4556 IU/L. Coombs direct and indirect, Hb Chromatography, and iron studies were unremarkable. JAK2 mutation was not detected.

**Figure 1:**
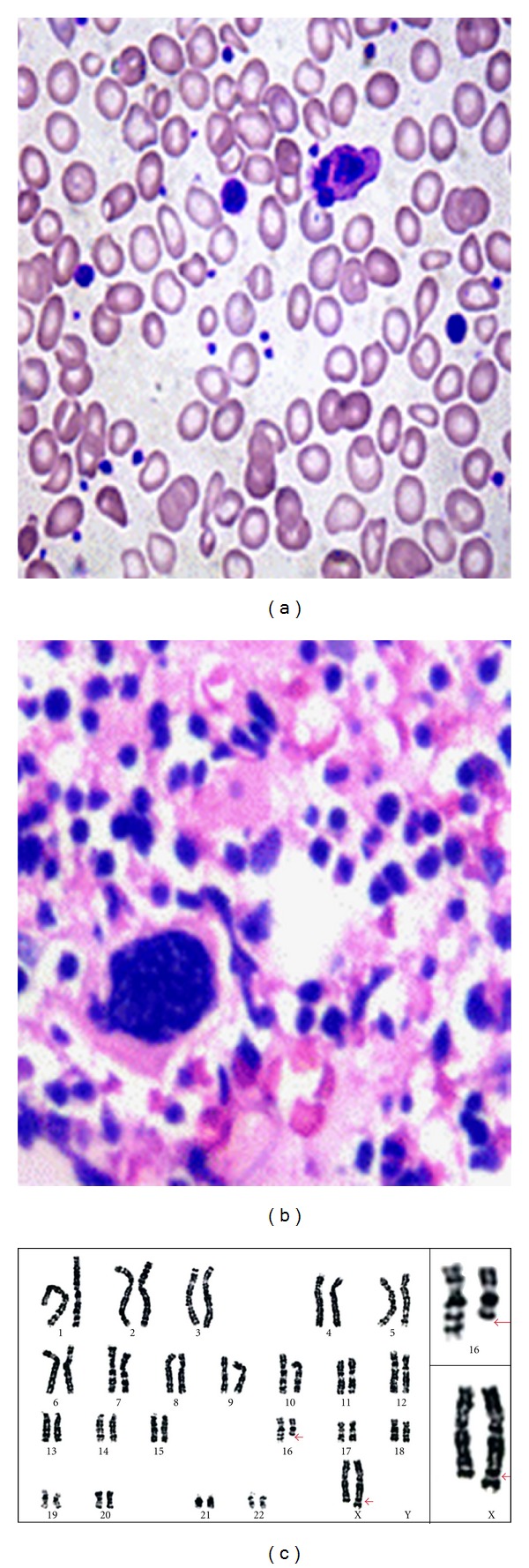
(a) blood smear illustrating tear drop poikilocytes and giant platelets. (b) BM biopsy showing an atypical megakaryocyte. H&E 45x. (c) Karyotypic analysis showing an abnormal clone with 46,X,t(X;16)(q28;q22).

 Peripheral smear demonstrated severe anisopoikilocytosis with oval macrocytes and hypersegmented neutrophils. Platelets were increased in number, exhibiting marked anisocytosis with many giant forms (Figure 1(a)). Bone marrow (BM) evaluation showed atypical megakaryocytes (Figure 1(b)) in a backdrop of megaloblastic erythroid hyperplasia. Reticulin fibrosis grade 4/4 with osteosclerosis and grossly distended marrow sinusoids were apparent. Marrow features were consistent with fibrotic stage of PMF. Karyotypic analysis revealed 46,X,t (X;16)(q28;q22)/46,XX. There was an abnormal clone with translocation of segment q22 of chromosome 16 to q28 region of X chromosome (Figure 1(c)). Intramuscular injections of vitamin B_12_ largely alleviated her symptoms. Hb improved to 12.1 g/dL and bilirubin values returned to normal. She was followed for four months through which her condition remained steady.

## 3. Case 2

**Figure 2:**
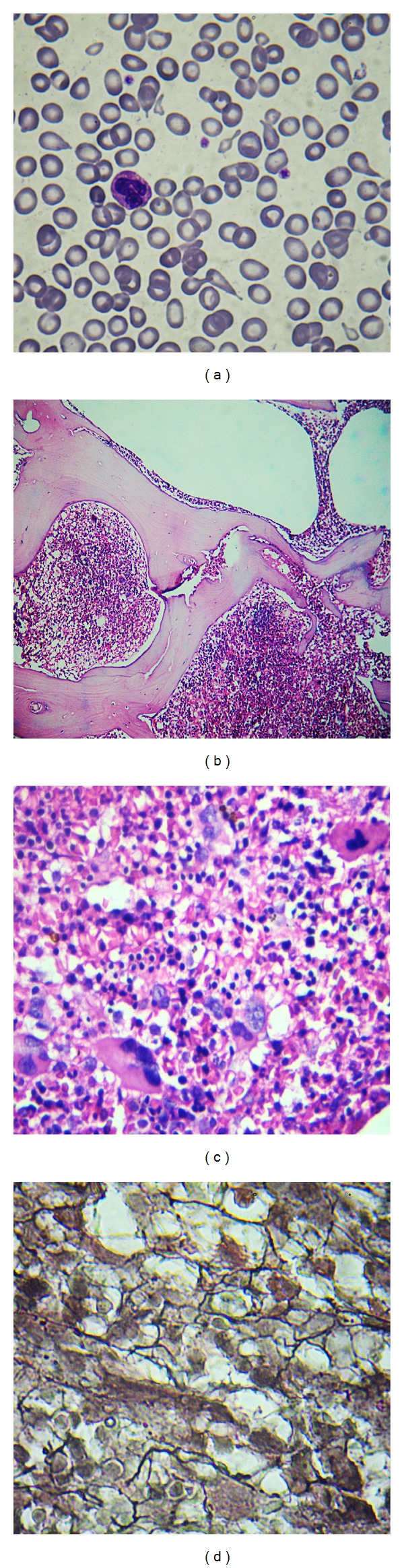
(a) Blood smear displaying anisocytosis and “tear drop” red cells. (b) BM biopsy showing hypercellular areas and distended marrow sinusoids. Megakaryocytes appear prominent even at low magnification. H&E 4x scanner. (c) BM biopsy illustrating increased number of atypical megakaryocytes against a hypercellular background. The “cloudy” nature of megakaryocyte nuclei is evident. H&E 45x. (d) grade 2/4 reticulin fibrosis highlighted by silver staining.

 A 53-year-old male was referred for persistent anemia during the past three years. Physical examination was unremarkable except for glossitis. Abdominal scan was normal. His initial investigations revealed Hb 5.8 g/dL, Hct 18.2%, MCV 95 fl, RBC 1.8 × 10^9^/L, reticulocytes 1.2%, WBC 36 × 10^9^/L with absolute neutrophil count 17.6 × 10^9^/L, platelets 76 × 10^9^/L. His serum folate was 2.1 ng/mL, vitamin B_12_ 380 pg/mL, iron 14 *μ*g/dL, ferritin 14.3 ng/mL, transferrin saturation 10%, uric acid 9.2 mg/dL, and LDH 356 IU/L. Blood smear showed moderate anisopoikilocytosis, tear drop cells, neutrophil leukocytosis, and thrombocytopenia (Figure 2(a)). There was no history of prior gastrointestinal surgery. Cause of anemia was initially attributed to the combined deficiencies of iron and folic acid.

A month following red cell transfusions and treatment of the deficiencies, blood counts were as follows: Hb 10.5 g/dL, WBC 37.5 × 10^9^/L, platelets 75 × 10^9^/L, and reticulocytes 4.8%. BM biopsy revealed mostly hypercellular areas with increased M : E ratio. Megakaryocytes were increased in number, exhibiting highly pleomorphic nuclei and bizarre forms. Many nuclei had cloudy, balloon-like appearance. Reticulin fibrosis of grade 2/4 along with few distended marrow sinusoids was *evident *(Figures 2(b), 2(c), and 2(d)). These features characterized the cellular phase of PMF, presenting with combined deficiencies of iron and folic acid. JAK2 mutation was negative and karyotype was normal. This case was referred to oncology for further management of MPN.

## 4. Case 3

**Figure 3:**
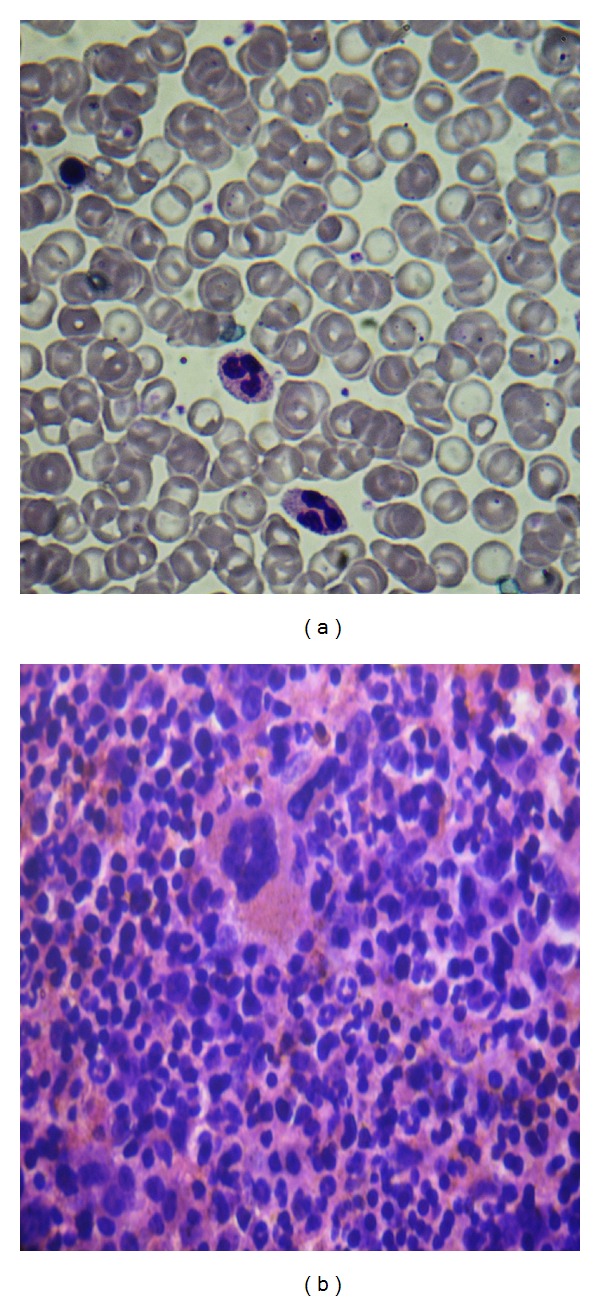
(a) blood smear displaying hypochromic red cells and increased neutrophils. A nucleated red cell is also seen. (b) Hypercellular marrow with obliteration of fat spaces. H&E 45x.

A 60-year-old male came with complaints of dizziness and tingling sensation in his fingers since three years, which had worsened during the past 6 months. He had suffered a cerebrovascular accident before one year. Although his earlier Hb values varied between 16.2 to 17.3 g/dL, a diagnosis of PV was not cited in his medical records. Spleen was palpable six centimeters below the left costal margin. His blood counts were as follows: Hb 16.3 g/dL, Hct 53.4%, MCV 68.5 fl, MCH 20.7 pg, RBC 8.34 × 10^9^/L, reticulocytes 1.1%, WBC 14.2 × 10^9^/L with 75% neutrophils, platelets 581 × 10^9^/L. Peripheral smear showed microcytic, hypochromic red cells with neutrophilia and thrombocytosis (Figure 3(a)). Serum ferritin was 12.4 ng/mL, iron 22 *μ*g/dL, transferrin saturation 5.2% suggesting iron deficiency. Vitamin B_12_ was 1447 pg/mL, LDH was 332 IU/L, and Hb chromatography confirmed a normal adult pattern. Serum erythropoietin level was within normal (45 U/L). JAK2 V617F was positive by PCR. BM was significantly hypercellular, with obliteration of fat spaces (Figure 3(b)) and trilineage hyperplasia. There was no evidence of reticulin or collagen fibrosis. These features confirmed the diagnosis of PV, manifesting with secondary iron deficiency. 

## 5. Discussion 

Three cases of Philadelphia-negative MPN, manifesting as longstanding nutritional deficiencies, are presented. All three patients were moderately built and nourished, with no history of prior gastrointestinal surgery.

*Case *1 illustrates PMF presenting as recurrent nutritional deficiency anemia. As nutritional deficiency is widely prevalent in India, further investigations to unveil the cause of anemia were not pursued for long. Her fundamental problem was overlooked for more than two years. This case also illustrates the importance of considering PMF in young patients. Unconjugated hyperbilirubinemia was attributed to ineffective hematopoiesis consequent to vitamin B_12_ deficiency. Translocations involving X chromosome have previously been reported in ET, PV, and unclassifiable MPN [7–9].

*Case *2 illustrates PMF presenting as longstanding anemia associated with combined nutritional deficiencies. There was no organomegaly, and JAK2 mutation was not detected. Inadequate response to treatment, neutrophil leukocytosis, numerous tear drop poikilocytes, and elevated uric acid raised a suspicion of PMF. 

*Case *3 signifies PV presenting with normal Hct and normal erythropoietin levels. Although PV commonly presents with elevated Hct, this concept is so ingrained that the possibility of PV is not thought of unless Hct is obviously high. Occurrence of iron deficiency at presentation can lower the Hb to near normal levels, thereby masking PV. In such situations, marked erythrocytosis is an important hint signifying the likelihood of PV. Appropriate evaluation of red cell parameters can therefore assist in recognizing PV presenting with near normal Hb. 

 Deficiencies of iron, folic acid, and vitamin B_12_, alone or in combination, have been reported in association with MPN [4–6]. In identifying subtle deficiency of vitamin B_12_ and folate, homocysteine and methyl malnoic acid (MMA) are proven to be more sensitive than serum cobalamin and folate. On the basis of measuring these metabolites before and after supplementation trials, Faurschou et al. [10] found a high incidence of both vitamin B_12_ and folate deficiency in myeloproliferative disorders. 

 In patients with myeloproliferative disorders, normal to high serum vitamin B_12_ concentrations have often been reported [11]. Hypervitaminemia B_12_ can sometimes paradoxically be accompanied by signs of deficiency reflecting a functional deficit related to defects in tissue uptake and action of vitamin B_12_. In a cross sectional observatory study of 33 patients with myeloproliferative neoplasms, Gauchan et al. [12] have reported normal to elevated serum vitamin B_12_ levels in all patients. However, 9 patients (27.3%) also exhibited elevated serum MMA, suggesting an occult cobalamin deficiency. In these patients, elevated levels of serum vitamin B_12_ actually masked the true deficiency. 

 Deficiencies of iron, vitamin B_12_, and folic acid are widely prevalent affecting millions of people, particularly in developing countries [13]. Prevalence of vitamin B_12_ and folic acid deficiencies is reported to be 33% and 6.8% in India [14], 51% and 17% in Zimbabwe [15], 56% and 8% in Pakistan respectively [16]. These deficiencies most frequently result from lack of proper nutrition. 

 In developing countries where nutritional deficiencies are prevalent, their coexistence with MPN is even more likely. In such situations where both disorders coexist, MPN is frequently unnoticed. Diagnosis of MPN is an elaborate task, requiring BM evaluation and molecular markers. Nevertheless, diagnosis of nutritional deficiencies is generally straightforward. Besides, patients show noticeable clinical improvement after treatment of deficiency states [6]. So, further investigations are usually held back and diagnostic efforts come to a standstill. Unless the anemia is considerably longstanding, BM evaluation is not thought of. As a result, many cases of MPN go undetected for long. Lack of regular followup and inadequate documentation further delays their diagnosis. 

 Nutritional deficiencies exhibit predominant anisopoikilocytosis, thereby masking the characteristic features of MPN, especially PMF. Occurrence of basophilia, tear drop poikilocytes, nucleated red cells, early myeloid precursors, giant platelets, agranular platelets, and megakaryocyte fragments in the peripheral blood smear are important clues pointing towards a primary myeloid neoplasm [17]. BM histology remains the mainstay in diagnosis of MPN, particularly in JAK2-negative cases and in situations where molecular genetics are not readily accessible. Suspecting an underlying MPN even in the context of nutritional deficiency and performing a BM biopsy is the key step in identifying these hidden disorders.
